# Clinicopathological characteristics and treatment of patients with high-grade endometrial stromal sarcoma: A retrospective study of 40 cases

**DOI:** 10.1097/MD.0000000000028490

**Published:** 2022-01-14

**Authors:** Huimin Bai, Fang Yuan, Bing Liang, Hengzi Sun, Yutao Gao, Mulan Jin, Xiaoming Xing

**Affiliations:** a Department of Obstetrics and Gynecology, Beijing Chao-Yang Hospital, Capital Medical University, Beijing, China; b Department of Obstetrics and Gynecology, the Affiliated Hospital Qingdao University, Qingdao, China; c National Cancer Center/National Clinical Research Center for Cancer/Cancer Hospital & Shenzhen Hospital, Chinese Academy of Medical Sciences and Peking Union Medical College, China; d Department of Pathology, Beijing Chao-Yang Hospital, Capital Medical University, Beijing, China; e Department of Pathology, the Affiliated Hospital Qingdao University, Qingdao, China.

**Keywords:** clinicopathological characteristics, FIGO stage, HG-ESS, high-grade endometrial stromal sarcoma, prognosis, treatment

## Abstract

To investigate the clinicopathological characteristics of patients with high-grade endometrial stromal sarcoma (HG-ESS).

The clinicopathological characteristics, treatments, and prognostic information of consecutive HG-ESS patients were collected from medical records and then evaluated.

A total of 40 women were included in the analysis. The immunohistochemical profiles indicated that HG-ESS tumors tend to be locally or weakly positive for vimentin (100%) and CD10 (72.0%) but mostly negative for desmin (7.7%) and AE1/AE3 (9.1%). The progression-free survival intervals and the clinical benefit rates of patients receiving radiotherapy and/or chemotherapy were slightly longer and higher than those receiving simple observation (progression-free survival: 6 and 5 months vs 2 months; clinical benefit rate: 83.3% and 75.0% vs 28.6%). The 1-year disease-specific survival (DSS) rate was 62.7%. Tumor size, myometrial invasion, lymphovascular space invasion, cervical involvement, Federation International of Gynecology and Obstetrics (FIGO) stage, and residual disease all significantly affected the DSS rate (*P* < .001, =.002, <.001, =.004, <.001, and <.001, respectively). For patients with stage I disease, the 1-year DSS rate was as high as 91.7%, in contrast to 66.7%, 26.7%, and 0% for those with stage II, III, and IV disease, respectively.

HG-ESS is associated with an adverse prognosis. FIGO stage could effectively predict the prognosis of patients with this lethal disease. Immunohistochemical markers, vimentin+/CD10+ (local or very weak), in combination with desmin-/AE1/AE3-, may be helpful for improving the diagnostic accuracy of this lethal condition. The therapeutic roles of adjuvant chemotherapy and radiotherapy warrant further investigation.

## 1 Introduction

Endometrial stromal sarcoma (ESS) is a rare type of uterine malignancy that develops from endometrial connective tissue, accounting for only 0.2% to 1% of all uterine malignancies and 6% to 20% of uterine sarcomas.^[1,2]^ ESS is classified into 4 subtypes according to the most recent World Health Organization classification: endometrial stromal nodule, low-grade endometrial stromal sarcoma (LG-ESS), high-grade endometrial stromal sarcoma (HG-ESS), and undifferentiated uterine sarcoma.^[3,4]^ HG-ESS tumors tend to exhibit extensive invasion, are composed of round cells with high mitotic activity (more than 10 mitoses per 10 high-power fields), and lack estrogen receptor (ER) and progesterone receptor expression. Compared with LG-ESS, which usually exhibits a slow clinical course, HG-ESS behaves much more aggressively and is associated with a significantly poorer prognosis, which has been demonstrated in several small case series^[5–7]^ and by our previous studies.^[8,9]^ Thus, these 2 distinct entities should be treated differently.

The optimal treatment for HG-ESS has not yet been established, and there have been very few reports on the effectiveness of implemented treatment strategies, mainly due to its rarity. Of the few previous studies focusing on HG-ESS, most are isolated case reports or include low-grade tumors and other histological uterine sarcoma subtypes.^[6,9–13]^ To date, only 3 relatively large series^[14,15]^ have been reported in the literature. Zhang et al^[16]^ compiled 40 patients with HG-ESS—a sample size as large as this analysis. Nonetheless, the clinicopathological characteristics of HG-ESS remain poorly defined. In this study, HG-ESS patients from 3e academic hospitals were included, with the goal of providing useful information to manage this uncommon yet highly lethal tumor.

## 2 Materials and methods

The medical records of patients diagnosed with and treated for HG-ESS at 3 hospitals from January 1994 to December 2014 were collected and reviewed. Patient records from the following hospitals were included: Beijing Chao-Yang Hospital, Capital Medical University; the Affiliated Hospital of Medical College Qingdao University; and the National Cancer Center/National Clinical Research Center for Cancer/Cancer Hospital & Shenzhen Hospital, Chinese Academy of Medical Sciences and Peking Union Medical College. All consecutive patients who underwent surgery and had complete surgical and pathology reports were included in the analysis, while patients who had insufficient data or were lost to follow-up within 1 month after surgery were excluded. Patient information, including demographic and pathological characteristics, response to treatment, and disease status at the date of last contact, was collected and evaluated.

The initial surgical procedure and subsequent adjuvant treatments were performed as previously described.^[8,9]^ Briefly, the initial surgical procedure consisted of total hysterectomy and bilateral adnexectomy in most cases. Staging lymphadenectomies were performed, generally depending on the extent of disease, the institutional practice at the time of surgery, and the patient's potential tolerance to surgery. All pathological slides were independently reviewed according to the 2014 WHO Classification^[4]^ by 2 pathologists with extensive backgrounds in gynecological pathology who were blinded to patient outcomes.

If any differences were noted between the 2 independent evaluations, a new consensus evaluation was conducted. Tumor size was determined by the largest tumor diameter. Macroscopic residual tumors were defined as any observable disease within the abdominopelvic cavity. Tumor staging was retrospectively assigned according to the 2009 Federation International of Gynecology and Obstetrics (FIGO) staging system. For patients who underwent incomplete staging surgery, the stage was assessed based on the operative records and available pathological findings, with unevaluated areas regarded as negative for metastatic lesions.^[8,17]^

Adjuvant treatment was performed without well-defined protocols. The decision to perform chemotherapy and/or radiotherapy was based on the extent of disease, medical comorbidities, institutional practices, or the doctor's preference. The commonly used adjuvant chemotherapy regimens included the following: PEI (cis/carboplatin, epirubicin, and ifosfamide), PAC (cis/carboplatin, adriamycin, and cyclophosphamide), and VAC (vincristine, adriamycin, and cyclophosphamide). The details of these regimens have been described in our previous study.^[8]^ Pelvic radiotherapy with or without a vaginal boost was the most frequently performed. According to the site and extent of disease, brachytherapy and whole abdominal radiotherapy were also performed. The responses to systemic agents or radiotherapy was recorded according to version 1.1 of the Response Evaluation Criteria in Solid Tumors.^[18]^ The clinical benefit rate (CBR) was estimated as the rate of the response-evaluable courses of chemotherapy in achieving complete remission (CR), partial response (PR), or stable disease (SD).

After the completion of treatment, the women were followed-up monthly for the first 6 months, every 3 months for next 6 months, and then every 6 months thereafter. Efforts were made to contact patients by telephone or mail to obtain follow-up information in cases where regular follow-up information was not available. Disease progression was documented by histological evidence of disease on tumor biopsy and/or by the appearance of new lesions on imaging examinations. Progression-free survival (PFS) was calculated in months from the time of tumor response to the time of progressive disease (PD); women who were disease-free at the time of their last visit were censored. Disease-specific survival (DSS) was calculated in months from the date of the initial diagnosis to the date of death from the disease; patients who died from other conditions and survivors at the time of their last follow-up visit were censored.

Patient records and information were anonymized and de-identified prior to analysis; therefore, consent was not necessary. The study protocol was approved by the ethics committees at the Beijing Chao-Yang Hospital, and the Affiliated Hospital of Qingdao University.

### 2.1 Statistical analysis

All statistical analyses were performed using SAS Version 9.2 (SAS Institute, Cary, NC). All of the tests were 2-sided, and *P* < .05 was considered to be statistically significant. The CBRs between active agents were compared using Fisher exact test. The Wilcoxon rank sum test was performed to compare PFS rates. The Kaplan-Meier method was used for univariate analyses of PFS and DSS. Log rank test was used to compare different survival curves.

## 3 Results

During the study period, 246 consecutive ESS patients were treated at the 3 hospitals. A total of 206 (83.7%) patients were diagnosed with LG-ESS and excluded from the analysis. Therefore, a total of 40 (16.3%) HG-ESS cases were ultimately included for further analysis (Table 1). The median age of the patients at the initial diagnosis was 49.4 (range: 17–78) years. Pre-menopausal women accounted for 67.5% (27 cases) of the cases, and 22.5% (9 cases) were nulliparous. One (2.5%) patient had previously received hormonal treatment; however, no additional details were provided in her record. The most common presentation was metrorrhagia (65.0%), followed by pelvic pain or pelvic pressure (22.5%) and rapid leiomyoma growth (15.0%). Four (10%) asymptomatic patients were incidentally diagnosed on pelvic examination. Ill-defined uterine lesions and/or abdominopelvic cavity masses were detected in all 40 patients through physical and/or imaging examinations, such as pelvic ultrasonography, computed tomography (CT), and/or magnetic resonance imaging (MRI). In addition, through pre-operative lung CT or MRI scanning, pulmonary metastasis was identified in 7 patients. A pre-operative diagnosis of potential uterine sarcoma or ESS was made for 24 (60%) patients via diagnostic curettage (15 cases), biopsy of the mass prolapsing out of the cervix (5 cases), or intra-operative sharp-freezing section histological examination (4 cases).

**Table 1:** Demographic and clinical characteristics of the 40 patients with high-grade endometrial sarcoma (HG-ESS).

Parameter	Number of patient	Percent (%)
Study period	21 mos	
The first half of study period	14	35.0
The last half of study period	26	65.0
Age at diagnosis, (yrs; median, range)	49.4 ± 14.7 (17–78)	
≤49	25	
>49	15	
Menstruation status		
Pre-menopause	27	67.5
Postmenopause	13	32.5
Presentation		
Metrorrhagia	26	65.0
Pelvic pain or pelvic pressure	9	22.5
Rapid growth of leiomyoma	6	15.0
Absence of symptoms	4	10.0
Pre-operative CA-125 (U/mL)		
≤35	28	70.0
>35	9	22.5
Data not available	3	7.5
Primary surgery		
Cervical conization	1	2.5
Hysterectomy	39	97.5
BSO	38	95.0
Lymphadenectomy	23	57.5
Surgical approach		
Laparotomic	35	87.5
Laparoscopic	5	12.5
Adjacent treatment		
Hormone therapy	3	7.5
Chemotherapy	21	50.0
Radiotherapy	10	22.5
Observation	14	35.0
Disease status at completion of primary treatment		
CR	27	67.5
PR	1	2.5
SD	3	7.5
PD	9	22.5
Follow-up (mos; mean, range)	19.9 ± 31 (1–165)	
Status at the last contact		
NED	18	45.0
AWD	9	22.5
DOD	13	32.5

AWD = alive with disease, BSO = bilateral salpingo-oophorectomy, CR = complete remission, DOD = die of disease, NED = no evidence of disease, PD = progressive disease, PR = partial response, SD = stable disease.

Surgical resection of the primary uterine disease was performed in all 40 patients and total hysterectomy and bilateral salpingo-oophorectomy were performed in 38 patients (95%). One patient with sarcoma confined within the cervix received cervical cold knife conization with ovary-sparing procedures. The remaining patient underwent hysterectomy without oophorectomy. Both of these patients had a strong desire to retain fertility and/or ovarian function and refused additional radical surgical procedures under fully informed consent. Pelvic lymphadenectomy was performed in 23 (57.5%) women for complete disease staging. The mean number of lymph nodes (LNs) removed was 23.3 ± 10.2 per patient (range: 10–52). LN micrometastasis was identified in 3 (13.0%) patients. After the initial surgery, 9 patients had residual disease in the abdominopelvic cavity (2 cases), the lung (4 cases), or both (3 cases).

The pathological characteristics of the 40 patients are shown in Table 2. Primary tumors were located in the uterine cavity (26 cases), the cervix (4 cases), or both (10 cases). The mean tumor diameter was 8.4 ± 6.1 (range 1.5–30) cm. Extrauterine diseases was present in 16 (40.0%) women. The sites of extrauterine disease included the parametrium (7 cases), adnexa (6), lungs (7), omentum (6), LNs (3), appendix (2), kidneys (2), and bladder (1). The stage staging distribution was as follows: 24 cases were classified as stage I (60%), including 10 cases of stage Ia and 14 cases of stage Ib; 5 were classified as stage II (12.5%); 4 were classified as stage III (25%); and 7 were classified as stage IV (17.5%).

**Table 2:** Pathological characteristics of the 40 HG-ESS cases.

Parameter	Number of patient	Percent (%)
Tumor size (cm) (mean; range):	8.4 ± 6.1 (1.5–30)	
≤5 cm	19	47.5
>5 cm	21	52.5
Myometrial invasion		
≤50%	12	30.0
>50%	28	70.0
Cervical involvement		
+	14	35.0
–	26	65.0
LVSI		
+	15	37.5
–	25	62.5
Necrosis		
+	13	32.5
–	27	67.5
Extrauterine disease		
+	16	40
–	24	60
Residual disease after initial surgery		
+	5	12.5
–	35	87.5
FIGO stage		
I	17	42.5
II	6	15
III	10	25
IV	7	17.5

HG-ESS = high-grade endometrial sarcoma, LVSI = lymphovascular space invasion.

Immunohistochemical (IHC) staining was performed with specimens from 29 cases, and the profiles are summarized and shown in Table 3. The data indicated that HG-ESS tends to be generally weakly (<1%) or focally positive for vimentin (100%), with 72.0% positivity for CD10, and 38.1% positivity for smooth muscle actin, and it tends to be mostly negative for desmin (7.7%) and AE1/AE3 (9.1%). In addition, 33.3% and 20% of the tumors were weakly or focally positive for ER and PR, respectively. Other antigens were unevaluable, as very few patients were tested in our series. The diagnosis of HG-ESS was confirmed in all 40 patients through histopathological examination in combination with IHC profiles.

**Table 3:** Immunohistochemical staining profiles of the 29 HG-ESS patients.

Antigen	Positive number/number tested	Positive rate, %
Vimentin	15/15	100.0
Caldesmon	7/7	100.0
Actin	3/3	100.0
CD99	4/5	80.0
CD10	18/25	72.0
CD34	5/8	62.5
KI67 (>50%)	6/11	54.5
CK	2/4	50.0
SMA	8/21	38.1
PR	5/15	33.3
ER	3/15	20.0
HMB45	1/5	20.0
S-100	1/5	20.0
P53	1/6	16.7
AE1/AE3	1/11	9.1
Desmin	1/13	7.7
CD117	0/4	0
Inhibin	0/3	0

Most of the immunohistochemical positive staining was weak (<1%) or focal. HG-ESS = high-grade endometrial sarcoma.

Adjuvant therapy was administered to 26 (65.0%) patients, with 21 (52.5%) receiving chemotherapy, 9 (22.5%) receiving radiotherapy, and 3 (7.5%) receiving hormone therapy, either alone or in combination. The remaining 14 (35.0%) patients did not receive any adjuvant treatment (simple observation). After and during the initial treatment, 9 patients exhibited rapid PD and died of the disease. PR and SD were achieved in 1 and 3 cases, respectively. The remaining 27 cases achieved CR.

The mean follow-up period was 19.9 months. During the study period, 10 patients who achieved CR after the initial treatment developed recurrence. The median relapse interval was 7 months. One patient exhibited an extraordinarily long PFS interval (70 months). The sites of invasion and metastasis included the abdominopelvic cavity (8 cases), lungs (3 cases), vaginal stump (2 cases), intestinal tract (2 cases), and vulva (1 case). Palliative treatment was administered, and only 1 patient achieved CR again through surgical resection and cis/carboplatin, epirubicin, and ifosfamide chemotherapy. SD was achieved in 5 patients. The remaining 4 women exhibited PD and died of multiple metastases. In total of 13 (32.5%) patients died of the disease, with a median DSS interval of 3 (range: 1–14) months. Eighteen (45.0%) patients survived without any evidence of tumor at the time of the last visit. The remaining 9 (22.5%) patients were living with the disease, including 7 with end-stage sarcoma.

**Figure 1:**
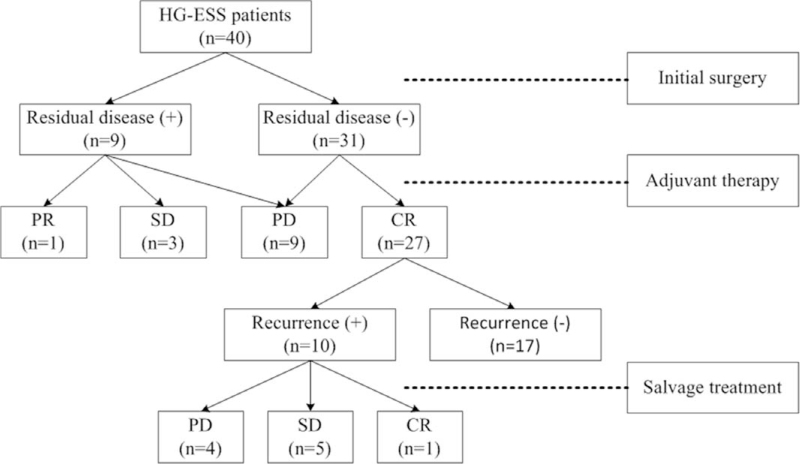
Patients’ response to treatment. CR = complete remission, HG-ESS = high-grade endometrial stromal sarcoma, PD = progressive disease, PR = partial response, SD = stable disease.

The treatment response is summarized in Figure 1. The response rates and duration of different adjuvant treatment modalities among the patients (19 cases, including 9 cases with residual disease after initial surgery and 10 cases with recurrent disease) with measurable disease were evaluated, and the results are shown in Table 4. The PFS intervals and CBRs of patients receiving radiotherapy and/or chemotherapy were slightly longer and higher, respectively, than those of patients undergoing simple observation (PFS: 6 and 5 months vs 2 months; CBR: 83.3% and 75.0% vs 28.6%). The effect of hormone therapy could not be evaluated since only 1 patient with measurable disease received this therapeutic modality in this analysis. No serious adverse events were found to be associated with chemotherapy, radiotherapy, or hormone therapy.

**Table 4:** Response rate and duration of different adjuvant treatment modalities for HG-ESS patients with measurable disease.

		Response (n)								
Adjuvant treatment	Patients (n)	CR	PR	SD	PD	UE	CBR (%)	*P* value^∗^	PFS [mos: median (range)]	*P* value^†^
Chemotherapy	10	0	1	5	2	2	75.0	.132	5 ± 1.5 (3–8)	.632
Radiotherapy	6	3	0	2	1	0	83.3	.103	6 ± 1.3 (3–9)	.536
Hormone therapy	1	0	0	1	0	0	100	.375	6	.541
None	7	0	0	2	5	0	28.6	Reference	2 ± 1.1 (0–5)	Reference
Total	19	3	1	8	7	0	63.2	–	3 ± 1.1 (0–9)	–

CBR = clinical benefit rate = (CR + PR + SD)/(no. of evaluable courses), CR = complete remission, HG-ESS = high-grade endometrial sarcoma, PD = progressive disease, PFS = progression-free survival, PR = partial response, SD = stable disease, UE = unevaluable.

∗ Fisher exact test.

† Log-rank test.

**Figure 2:**
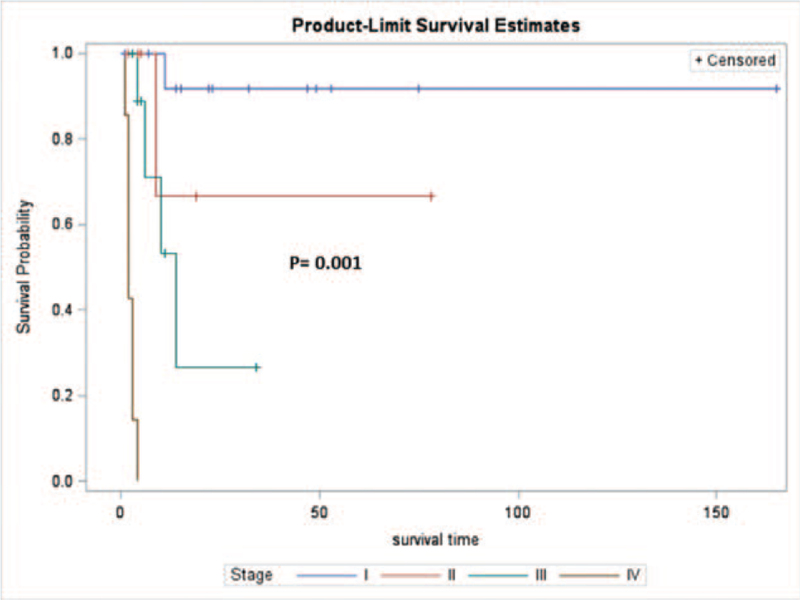
FIGO stage was an independent predictor of survival for patients with HG-ESS (*P* = .001). HG-ESS = high-grade endometrial sarcoma.

For the entire series, the 1-year DSS was 62.7%. The log-rank test revealed that tumor size, lymphovascular space invasion (LVSI), myometrial invasion, cervical involvement, FIGO stage, and residual disease were the significant adverse prognostic factors of HG-ESS (*P* < .001, *P* < .001, *P* = .002, *P* = .004, *P* < .001, and *P* < .001, respectively, Table 5). The FIGO stage was identified as an independent predictor for survival (*P* = .001, Fig. 2). For patients with stage I disease, the 1-year DSS rate was as high as 91.7%. In contrast, it was 66.7%, 26.7%, and 0% for those with stage II, III, and IV disease, respectively.

**Table 5:** Survival predictors for patients with HG-ESS.

Parameter	One-year DSS (%)	*P* value^∗^	*P* value^†^ [HR (95%CIs)]
Study period			
The first half of study period	59.9	.366	
The last half of study period	59.6		
Age			
≤47	67.4	.696	
>47	56.8		
Menopausal status			
Pre-menopausal	63.8	.261	
Postmenopausal	60.6		
BSO			
+	64.1	.105	
–	50.0		
Lymphadenectomy			
+	58.3	.901	
–	68.1		
Residual disease after initial surgery			
+	0	<.001	.913
–	74.9		
Myometrial invasion			
≤50%	90.9	.002	.668
>50%	50.6		
Tumor size			
≤5 cm	74.9	.004	.387
>5 cm	49.9		
Cervical involvement			
+	30.7	.004	.164
–	76.6		
LVSI			
+	24.0	<.001	.103
–	87.5		
FIGO stage			
I	91.7	<.001	.001 [4.317 (1.500–12.423)]
II	66.7		
III	26.7		
IV	0		

BSO = bilateral salpingo-oophorectomy, CI = confidence interval, DSS = disease-specific survival, HG-ESS = high-grade endometrial sarcoma, HR = hazard ratio, LVSI = lymphovascular space invasion.

∗ Log-rank test.

† Cox proportional hazards model.

## 4 Discussion

Because of its very low incidence, very few reports in the literature have described the medical treatment of HG-ESS. Therefore, reaching a consensus regarding the best management of this disease is very difficult. Based on our data, HG-ESS represents 16.3% of all ESS cases, which is within the range of previous reports (5%–49%).^[6,7,11,15,19–21]^ HG-ESS is well known for its aggressive nature and association with a poor clinical outcome.^[6,9,14,15,21,22]^ In the present study, the 1-year DSS rate was as low as 62.7%, and the mean DSS time was only approximately 1.5 years.

First, the present study demonstrated that tumor size, myometrial invasion, cervical involvement, LVSI, and FIGO stage were significant predictors of survival for patients with HG-ESS according to the log-rank analysis. A study by Ríos et al^[14]^ also suggested that tumor size, LVSI, and myometrial invasion were possibly related to a worse outcome. However, statistical conclusions regarding these prognostic factors could not be made due to the very small sample size (13 cases of HG-ESS). It was noted that the presence of LVSI is associated with LN metastases, that a larger tumor size is responsible for a more advanced stage, and that the FIGO stage is the main prognostic predictor in many types of uterine carcinomas.^[8,23–25]^ Based on our data, the 2009 FIGO staging system was independently associated with patient survival. For patients with stage I disease, the 1-year DSS rate was as high as 91.7%. In contrast, it was 66.7%, 26.7%, and 0% for those with stage II, III, and IV disease, respectively.

Therefore, early diagnosis is urgently needed for this lethal tumor. However, specific diagnostic methods for HG-ESS are still not available. Based on our data, most patients (90%) presented with non-specific symptoms such as metrorrhagia, rapid leiomyoma growth, and pelvic pain or pelvic pressure. These symptoms suggested the potential presence of HG-ESS or other types of uterine malignancies. Further examinations were consequently required. Pre-operative imaging examinations, such as MRI, CT, and positron emission tomography-CT, were useful for detecting potential extrauterine or distant disease. Diagnostic curettage was recommended for patients with metrorrhagia or abnormal endometrial thickness. Intra-operative sharp-freezing section histological examinations were also helpful for improving the pre-operative diagnosis of HG-ESS.

Tumor cells of HG-ESS lacking stromal differentiation, making histological diagnosis of this disease difficult. IHC staining is often used as an adjunct to morphological studies of HG-ESS and other types of uterine mesenchymal lesions. However, hardly any immunophenotype studies have focused on HG-ESS, the few studies often included LG-ESS tumors and other histological subtypes of uterine sarcoma. Vimentin, the primary intermediate filament protein of mesenchymal cells and tissues, has been regarded as a classical epithelial-to-mesenchymal transition biomarker.^[26]^ Increased vimentin expression has been reported in various tumor cell lines and tissues, including endometrial cancer.^[27]^ The results of this study indicated that HG-ESS tumors were generally positive for vimentin (100%). CD10 is a cell membrane metallopeptidase. Jana et al^[28]^ demonstrated that the stromal expression of CD10 in breast cancer is correlated with high-grade tumors, a poor prognosis, and ER negativity. CD10 is also usually expressed in endometrial stroma and endometrial stromal neoplasms.^[29–31]^ The positivity rate of CD10 in HG-ESS tumors determined in this analysis was 72.0%. Chu et al^[32]^ demonstrated that CD10 can be used to differentiate ESS from uterine cellular leiomyoma and leiomyosarcoma. In contrast, desmin is usually positive in smooth muscle cells but negative in endometrial stroma.^[33]^ AE1/AE3 are expressed during epithelial cell differentiation and are specific epithelial markers.^[34]^ The expression of desmin and AE1/AE3 is generally negative in HG-ESS, but in this study, the positivity rates of desmin and AE1/AE3 expression were 7.7% and 9.1%, respectively. IHC markers such as vimentin+/CD10+ (local or very weak), in combination with desmin-/AE1/AE3-, might be helpful for improving the diagnostic accuracy of this rare yet lethal condition.

Several characteristic genetic mutations identified in HG-ESS tumors might also be helpful to improve the diagnostic accuracy. Lee et al^[35]^ demonstrated that HG-ESS tumors typically harbor (10;17) (q22;p13) with a YWHAE–FAM22 gene fusion, distinct from low-grade tumors. In contrast, Hoang et al^[36]^ described the recent characterization of HG-ESS tumors with t(10;17) (q22;p13) resulting in YWHAE–NUTM2A/B fusion. YWHAE–NUTM2 fusion in tumors has been reported to be associated with BCOR mRNA upregulation.^[37]^ Lewis et al^[38]^ identified ZC3H7B–BCOR fusion in all HG-ESS tumors through fluorescence in situ hybridization and targeted RNA sequencing.

Currently, surgery is still considered to be the primary treatment modality for HG-ESS. All patients in the present series underwent surgical resection. Our data demonstrated that hysterectomy and resection of macroscopic lesions can improve the prognosis of this lethal disease. HG-ESS cells spread primarily through the bloodstream rather than the lymphatic vessels.^[14]^ According to our data, lymphadenectomy had no significant impact on the patients’ DSS, and no nodal relapse was identified in the present study; the patients seemed to benefit little from this procedure. However, 13.0% of the patients were found to have of pelvic node micrometastasis in the initial surgery. Therefore, from our point of view, lymphadenectomy should still be conserved as a treatment option for this lethal disease, either to provide prognostic information or to guide postoperative treatment.

The literature contains little information related to adjuvant therapy for HG-ESS. Tanner et al^[15]^ demonstrated that gemcitabine–docetaxel and doxorubicin-based chemotherapy achieved objective but short-lived responses in 5 patients with measurable disease. Radiotherapy showed 100% local control in 8 patients who received pelvic radiotherapy after surgery.^[14]^ Based on our data, the clinical benefit rates and PFS intervals of patients with measurable diseases receiving chemotherapy and radiotherapy were slightly higher and longer than those receiving simple observation. Zhang et al^[16]^ also demonstrated that the combination of surgery with radiotherapy and chemotherapy may improve the PFS of patients with early-stage disease. However, definitive conclusions could not be drawn regarding the efficacy of these adjuvant modalities in treating HG-ESS. The patients who exhibited the rapid progression were less likely to receive adjuvant therapy because of rapid deterioration of their physical status. This selective difference may bias our conclusion to some extent. Further investigation is warranted to validate these results.

This analysis had several limitations associated with its retrospective design, such as potential referral bias and other types of selection bias. Nonetheless, the present study compiled 40 HG-ESS cases, making it one of largest case series on this rare condition. Additionally, the data in this study span the most recent 20 years and therefore provide the latest management insights for this disease.

In summary, HG-ESS is a distinct histological subtype of uterine sarcoma that is associated with an adverse prognosis. The FIGO staging system could effectively predict the prognosis of patients with this lethal disease. IHC markers, such as vimentin+/CD10+ (local or very weak) in combination with desmin-/AE1/AE3-, as well as several characteristic genetic mutation tests, may be helpful for improving the diagnostic accuracy of this lethal condition. The therapeutic roles of adjuvant chemotherapy and radiotherapy warrant further investigation.

## Author contributions

HMB: conception and design of the study, assembly, analysis and interpretation of the data, and manuscript writing. FY, LB, HZS, YTG, MLJ, and XMX: provision of study materials or patients, analysis and interpretation of the data.

**Conceptualization:** Huimin Bai.

**Data curation:** Huimin Bai, Bing Liang, Hengzi Sun, Yutao Gao, Mulan Jin, Xiaoming Xing.

**Formal analysis:** Huimin Bai, Bing Liang, Hengzi Sun, Yutao Gao, Mulan Jin, Xiaoming Xing.

**Funding acquisition:** Huimin Bai, Hengzi Sun, Xiaoming Xing.

**Investigation:** Huimin Bai, Fang Yuan, Bing Liang, Hengzi Sun, Yutao Gao, Mulan Jin.

**Methodology:** Huimin Bai, Yutao Gao, Mulan Jin, Xiaoming Xing.

**Project administration:** Huimin Bai.

**Resources:** Huimin Bai, Fang Yuan, Bing Liang.

**Software:** Huimin Bai.

**Supervision:** Huimin Bai.

**Validation:** Huimin Bai.

**Visualization:** Huimin Bai.

**Writing – original draft:** Huimin Bai.

**Writing – review & editing:** Huimin Bai.
